# Changes in Ion Transport across Biological Membranes Exposed to Particulate Matter

**DOI:** 10.3390/membranes13090763

**Published:** 2023-08-29

**Authors:** Jakub Hoser, Adrianna Dabrowska, Miroslaw Zajac, Piotr Bednarczyk

**Affiliations:** Department of Physics and Biophysics, Institute of Biology, Warsaw University of Life Sciences—SGGW, 159 Nowoursynowska St. 159, 02-776 Warsaw, Poland; jakub_hoser@sggw.edu.pl (J.H.); adrianna_dabrowska@sggw.edu.pl (A.D.); miroslaw_zajac@sggw.edu.pl (M.Z.)

**Keywords:** black lipid membrane, particulate matter, electrophysiology, gramicidin A channel

## Abstract

The cells of living organisms are surrounded by the biological membranes that form a barrier between the internal and external environment of the cells. Cell membranes serve as barriers and gatekeepers. They protect cells against the entry of undesirable substances and are the first line of interaction with foreign particles. Therefore, it is very important to understand how substances such as particulate matter (PM) interact with cell membranes. To investigate the effect of PM on the electrical properties of biological membranes, a series of experiments using a black lipid membrane (BLM) technique were performed. L-α-Phosphatidylcholine from soybean (azolectin) was used to create lipid bilayers. PM samples of different diameters (<4 (SRM-PM4.0) and <10 μm (SRM-PM10) were purchased from The National Institute of Standards and Technology (USA) to ensure the repeatability of the measurements. Lipid membranes with incorporated gramicidin A (5 pg/mL) ion channels were used to investigate the effect of PM on ion transport. The ionic current passing through the azolectin membranes was measured in ionic gradients (50/150 mM KCl on *cis/trans* side). In parallel, the electric membrane capacitance measurements, analysis of the conductance and reversal potential were performed. Our results have shown that PM at concentration range from 10 to 150 μg/mL reduced the basal ionic current at negative potentials while increased it at positive ones, indicating the interaction between lipids forming the membrane and PM. Additionally, PM decreased the gramicidin A channel activity. At the same time, the amplitude of channel openings as well as single channel conductance and reversal potential remained unchanged. Lastly, particulate matter at a concentration of 150 μg/mL did not affect the electric membrane capacity to any significant extent. Understanding the interaction between PM and biological membranes could aid in the search for effective cytoprotective strategies. Perhaps, by the use of an artificial system, we will learn to support the consequences of PM-induced damage.

## 1. Introduction

Since the industrial revolution, increasing amounts of undesirable substances having a serious impact on human health are being released into the atmosphere. Air pollution, a common phenomenon occurring all over the world, is one of the greatest environmental threats to public health. Particulate matter (PM) is considered to be the most harmful components of the pollution cocktail [1]. The problem of PM was raised for the first time in the 1960s by scientists and nature conservationists. Since then, initiatives have been taken to address this problem with the development of methods to monitor and predict PM levels and their health consequences. Currently, short- and long-term PM exposure is the cause of many diseases and a concern of scientists around the world [2].

Particulate matter is a complex mixture of various compounds including organics, ions and metals [3]. PM are classified using the Equivalent Aerodynamic Diameters (EAD) method into PM10 (diameter < 10 μm), PM2.5 (diameter < 2.5 μm), PM0.1 (diameter < 0.1 μm) [4]. The adverse health effects of particulate matter depend on physical characteristics (such as the number, particle size, surface, electrostatic properties), biological and chemical composition [5]

It is clearly evident that individual components of particulate matter alone or in mixtures with other pollutants can cause adverse biological responses and increase morbidity not only in humans [6]. Due to their small size (<10 μm), particulate matter can penetrate biological tissues, induce noxious effects such as disruptions of membranes and cytoskeletons, the generation of reactive oxygen species, among others [7,8]. PM10 deposits mainly in the upper airway and digestive system, while PM2.5 and PM0.1 can translocate across the air–blood barrier and enter the extrapulmonary organs [6,9]. These findings suggest that the studies of the interaction between cellular membranes and particulate matter represent an important problem. There are still very few data describing the interaction of ion channels with PM. To date, it was only reported for transient receptor potential (TRP) proteins [8] and CFTR channel [10]. However, to our knowledge, the impact of PM on biological membrane permeability has not yet been extensively studied yet.

Ion transport across biological membranes is essential for maintaining cellular homeostasis and regulating various cellular processes, such as cell signaling, energy metabolism and nutrient uptake [11]. One of the excellent techniques used to study the ion transport and electrophysiological properties of biological membranes is the black lipid membrane (BLM) technique [12].

BLMs are a type of artificial membrane used in various scientific and technological applications [13,14,15]. They consist of a thin, self-assembled lipid bilayer, separating two compartments (*cis/trans*) filled with electrolyte solutions. The BLM technique is widely used in various areas of research, including biophysics, electrophysiology and drug discovery [16]. BLMs are mostly used in the studies of ion channel functions, drug permeability assays, membrane–protein interactions, and in the exploration of general membrane behavior [17]. Here, we used the BLM technique to investigate the effects of different particulate matter sizes and concentrations on the biophysical properties of pure artificial membranes as well as their interaction with previously built-in channels formed by gramicidin A (gA) peptides.

Gramicidin A is a naturally occurring peptide that forms transmembrane channels by the transbilayer dimerization of two subunits [18]. The gA ion channels are selective for monovalent cations (H^+^, Na^+^, and K^+^) [19,20] and are one of the most studied and best characterized membrane bound proteins [14]. It was shown that gramicidin channels share structural features with real ion channels [21]. Therefore, they can be excellent models for transmembrane channels in artificial membranes [22].

The effect of particulate matter on the biophysical properties of biological membranes has not been fully described yet. In the present work, we analyze the ionic currents and membrane-capacity changes induced by the presence of PM. Our results show that particulate matter decreases basal ionic currents at negative potentials, while it increases them at positive ones. We also show that the presence of PM does not cause any statistically significant changes in electrical membrane capacitance. Moreover, PM inhibits the channels formed by gramicidin A, however with no effect on the conductance and reversal potential. Taken together, our results provide new insights into the biophysical properties of biological membranes and ion channels in the presence of PM.

## 2. Materials and Methods

### 2.1. Particulate Matter

To ensure the repeatability of biophysical and biochemical experiments, particulate matter (PM) samples (SRM-2786, with diameter < 4 μm (PM4.0) and SRM-2787, with diameter < 10 μm(PM10)) were purchased from the National Institute of Standards and Technology (NIST, Gaithersburg, MD, USA) and used as the reference material. The samples were composed of particulate matter collected by an air-intake filtration system from the exhibition center in Prague, Czech Republic. According to NIST, the PM samples used in our experiments represent the typical atmospheric particulate matter content found in urban areas. This includes the polycyclic aromatic hydrocarbons (PAHs), polybrominated diphenyl ethers (PBDE) and inorganic constituents (including metals, such as Al, Zn, Cd and ions carrying positive and negative charges) [23] that might affect the biophysical properties of membranes. PM samples were prepared according to the producer’s recommendations before use.

### 2.2. Chemicals

1,2-Diacyl-*sn*-glycero-3-phosphocholine (asolectin), n-decane and Gramicidin A were from MERCK, Darmstadt, Germany. Gramicidin A was dissolved in ethanol and incorporated into black lipid membranes at a final concentration of 5 pg/mL. All of the chemicals used were of analytical grade.

### 2.3. Cell Culture

Parts of our experiments were performed on the human bronchial epithelial cell line (16HBE14σ, MERCK, Darmstadt, Germany). Cells were cultured on T75 flasks (Nunc, Thermo Fisher) in Minimal Essential Medium (MEM; MERCK, Darmstadt, Germany) supplemented with 10% fetal bovine serum (FBS; Gibco, Thermo Fisher Scientific, Waltham, MA, USA) and antibiotics—100 U/mL penicillin and 100 mg/mL streptomycin (MERCK, Darmstadt, Germany). Cells were maintained within a humidified 5% CO_2_ atmosphere at 37 °C and reseeded when they reached 90% confluence.

To illustrate the effect of particulate matter (PM) on the human bronchial epithelium (16HBE14σ), the cells were grown in cell culture medium alone (control) or supplemented with PM. Cell morphology was viewed after 24, 48 and 72 h of incubation using an inverted optical microscope (Olympus CKX53, Olympus, Tokyo, Japan).

### 2.4. Black Lipid Membrane Technique

Asolectin dissolved in n-decane at a final concentration of 25 mg/mL was used as a model of the biological membrane and was applied using a polyethylene brush to the 250 μm diameter opening of the Teflon cup to separate two chambers (*cis/trans*). To improve the stability of the lipid bilayer, the outline of the aperture was coated with a lipid solution and N_2_-dried prior to bilayer formation. Overall, 1 mL quantities of the solutions containing 50/150 mM KCl (*cis/trans*) and 10 mM HEPES at pH = 7.2 were added to measurement chambers and stirred via magnetic stirrers. Silver-chloride (Ag/AgCl) electrodes were introduced into the chambers and connected to the BLM-120 amplifier (Bio-Logic, Seyssinet-Pariset, France). The electric signal was processed by the PowerLab 2/25 (ADInstruments, Sydney, Australia) converter, recorded using LabChart5 software and analyzed in the Clampfit 8 software. Experiments were performed in Faraday’s cage to prevent external electromagnetic interferences. All measurements were carried out at room temperature (25 °C).

Bilayer formation and thinning were monitored by the capacitance measurements and optical observations. The final accepted capacitance values ranged from 110 to 180 pF. Electrical connections were made by Ag/AgCl electrodes and agar salt bridges (3 M KCl) to minimize liquid junction potentials. Voltage was applied to the *cis* compartment and the *trans* compartment was grounded.

### 2.5. Data Analysis

Single-channel data were filtered at 500 Hz. The current was digitized at a sampling rate of 100 kHz. The data recordings illustrated are representatives of the most frequently observed currents under the given conditions. The conductance was calculated from the current–voltage relationship indicated by Ohm’s law. Reversal potential was calculated from the curve fitted to the measurement points (for the ion current equal to zero) [22,24]. Single-channel currents were recorded at different voltages in steps of 20 mV. The data are reported as mean value ± SD (SD, standard deviation).

## 3. Results

To study the impact of particulate matter (PM) on biological membranes, a number of cell cultures and electrophysiological experiments were performed. These included the cell culture and black lipid membrane (BLM) experiments.

### 3.1. Cell Morphology

**Figure 1:**
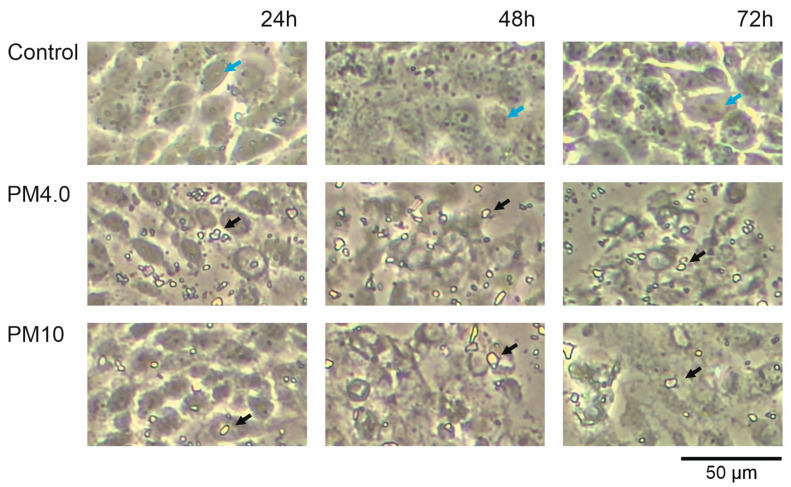
Human bronchial epithelial cells were incubated in cell culture medium alone and in the presence of PM4.0 and PM10 for 24 h, 48 h and 72 h, respectively. Fragments of PM particles (<4 μm and PM < 10 μm) deposited on HBE cells are clearly visible. Blue arrows indicate healthy cell examples and black arrows indicate chosen particulate matter (PM). The pictures were taken with the DLTX1080PCMOSHDU2SD camera (DELTA optical) placed in inverted optical microscope (Olympus).

The effect of different urban particulate matter sizes (PM4.0 and PM10) on the human bronchial epithelial line (16HBE14σ) morphology was visualized. Figure 1 presents the pictures of the cells incubated in the cell culture medium alone (control) and supplemented with PM4.0 and PM10 at a concentration of 50 µg/mL after 24, 48 and 72 h of incubation. The presence of PM (both sizes) caused the change in cell shapes and the increase in cell damage in a time-dependent manner. The incubation with PM samples resulted in an increased number of death cells and cellular debris observed in the medium as well as in the disruption of connections between neighboring cells.

### 3.2. Basis of the Measurements and Analysis

**Figure 2:**
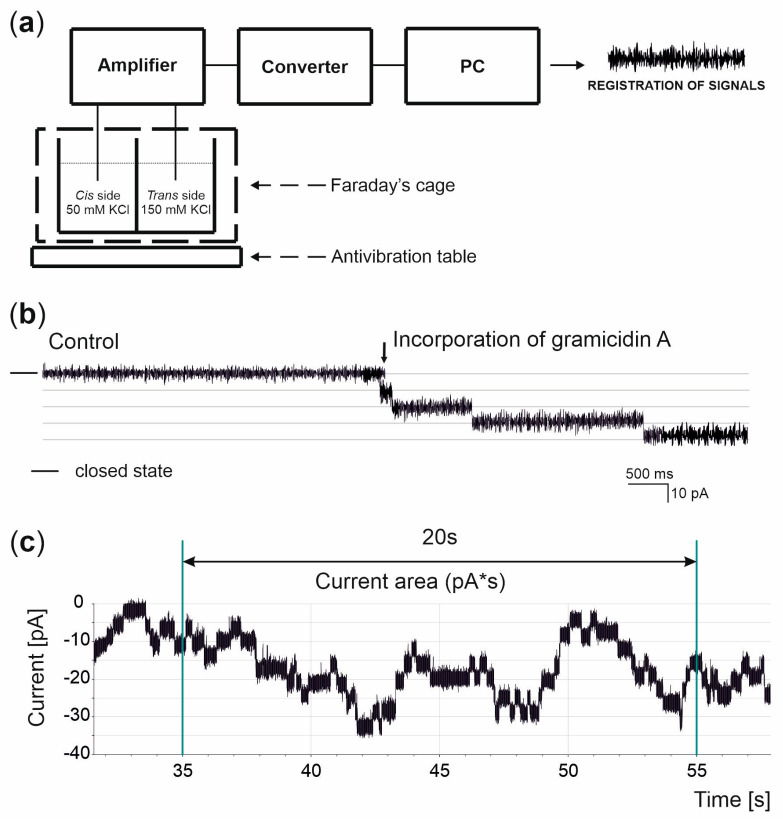
Black lipid membrane technique. (**a**) Scheme of the system used in black lipid membrane (BLM) experiments including antivibration table, Faraday’s cage, chamber with *cis/trans* side, amplifier, converter and PC. Buffers were at pH = 7.2 and *trans* side was grounded. (**b**) Representative recording in 50/150 mM KCl (*cis/trans*) gradient before and after incorporation of gramicidin A (arrow) at 0 mV—indicates the closed state. (**c**) Multi-channel recording in 50/150 mM KCl (*cis/trans*) gradient after incorporation of gA. The graph shows the method of ionic current area (pA*s) calculation over 20 s through lipid membrane with incorporated gramicidin A ion channel.

The main focus of our research was to investigate how PM affects biological membranes (lipid bilayers) and the gramicidin A model ion channel. Figure 2a presents the experimental setup used. An azolectin lipid bilayer with an electric capacity of approximately 120 pF separated two solutions (50/150 mM KCl (*cis/trans*). The basal currents observed across the pure lipid bilayer were in the range of 0.1 to 0.5 pA (depending on the voltage applied). Gramicidin A incorporation caused the increase in ionic currents passing through the lipid membrane (Figure 2b). To depict the ionic current changes caused by PM, the ionic current area was analyzed for 20 s current–time periods, as shown in Figure 2c.

### 3.3. Interaction of Particulate Matter with Lipid Membranes

**Figure 3:**
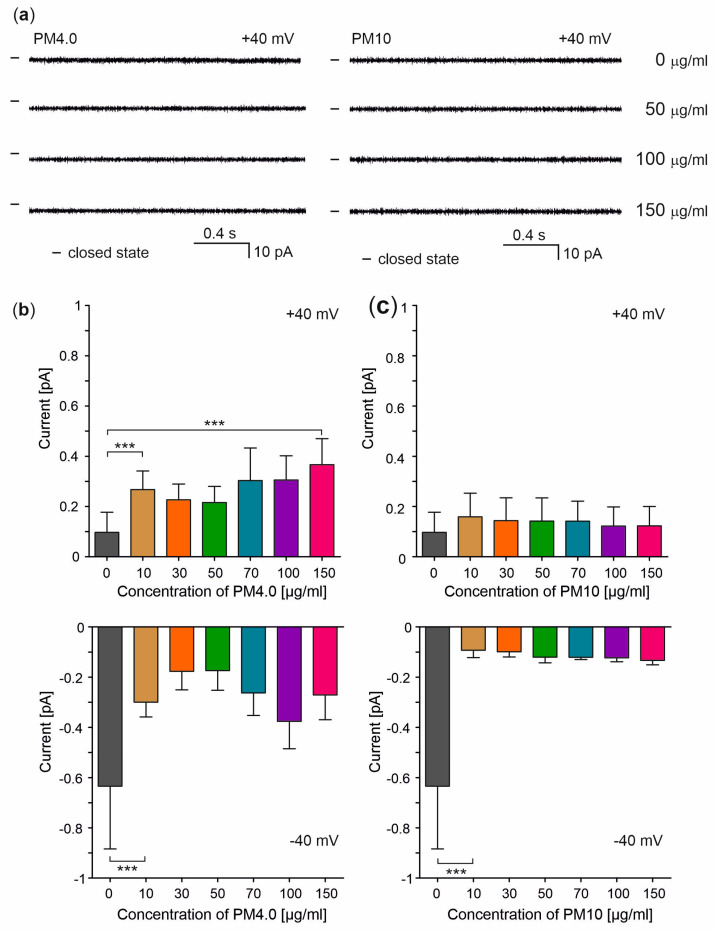
Effects of different PM concentrations on lipid membranes. (**a**) Registered current–time signals of ionic current flow through lipid bilayer membranes in control (0 μg/mL) and in the presence of 50, 100, 150 μg/mL PM4.0 and PM10 at a potential of +40 mV. (**b**,**c**) Analysis of the ionic current flow through lipid membrane at +40 and −40 mV without (control) and in the presence of PM4.0 and PM10 at concentrations of 10, 30, 50, 70, 100 and 150 μg/mL. The results are presented as mean ± SD, *n* = 5. Statistical significance was determined at *p* < 0.001 (***) using one-way ANOVA.

The presence of PM affected the electric parameters of the lipid bilayers. Interestingly, PM decreased the basal ionic currents at negative potentials while it increased them at positive ones (Figure 3). Surprisingly, the membrane disruption and irregularity of ion currents were not observed. Representative current–time traces and an analysis of the ionic current changes caused by different PM concentrations (0 to 150 μg/mL) at −40 and +40 mV potentials are shown in Figure 3a–c, respectively.

**Figure 4:**
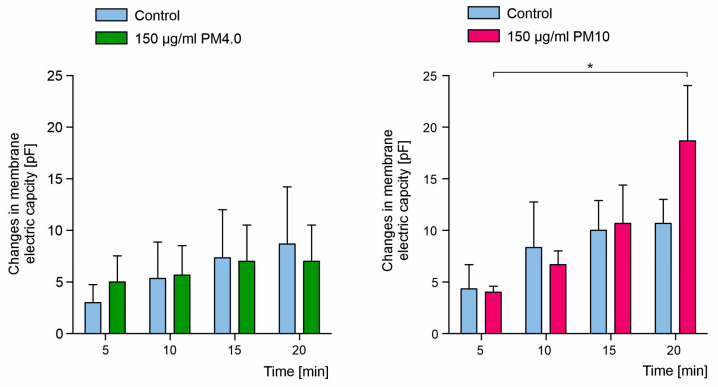
Electrical membrane capacitance changes in the presence of PM. Time-resolved effect of PM4.0 and PM10 (150 μg/mL) on electrical membrane capacitance changes. Results are presented as mean ± SD, *n* = 3. Statistical significance was determined at *p* < 0.05 (*) using one-way ANOVA.

In parallel, electrical membrane capacitance was measured. It is generally assumed that its changes may reflect the changes in lipid bilayer thickness. The electric capacity of lipid bilayers was measured in 5 min intervals before (control) and after the addition of PM4.0 and PM10 at a final concentration of 150 μg/mL (Figure 4, left and right panel, respectively). The presence of 150 μg/mL PM4.0 had no statistically significant effect on membrane capacitance. However, the addition of PM10 resulted in significant change in membrane capacitance observed after 15 min of incubation (Figure 4, right panel).

### 3.4. Interaction of Particulate Matter with Gramicidin A Channel

**Figure 5:**
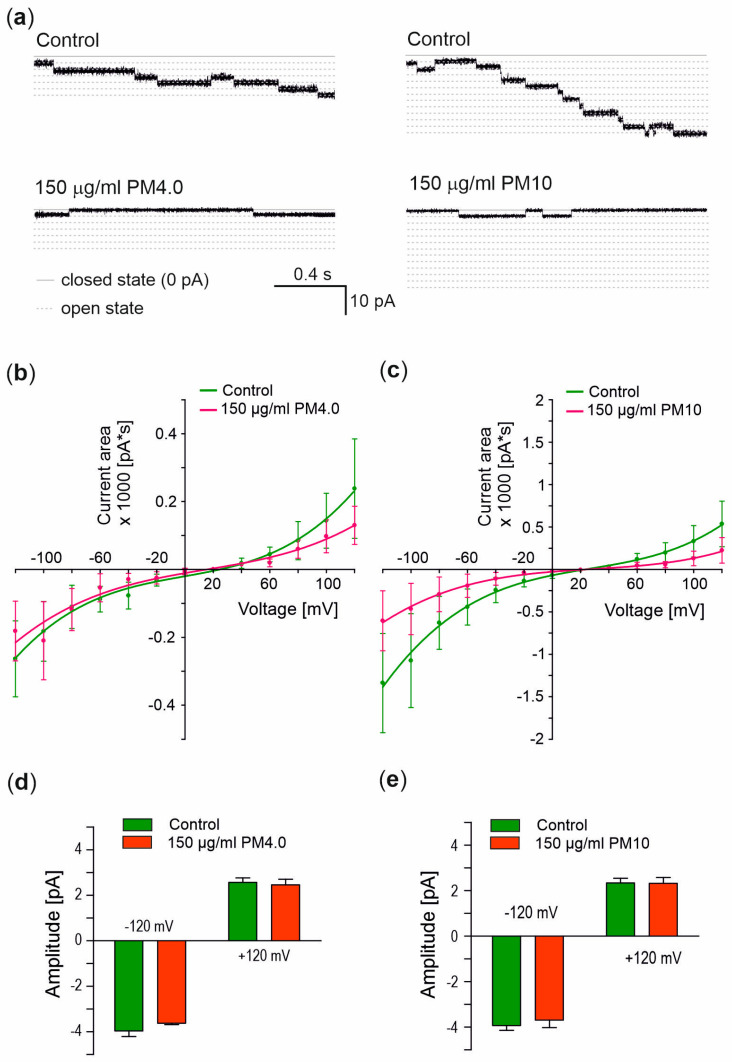
Effects of PM on gramicidin A channels. (**a**) Registered signals of ionic current flow through azolectin bilayer membrane with incorporated gramicidin A (5 pg/mL) channels at −120 mV with and without presence 150 μg/mL of PM. (**b**,**c**) Effect of particulate matter on ionic current area through azolectin bilayer membrane with incorporated gramicidin A (5 pg/mL) channels with and without the presence of 150 μg/mL PM4.0 (**b**) and PM10 (**c**). (**d**,**e**) Effect of the particulate matter on ionic current amplitude of single gramicidin A (5 pg/mL) channel in the presence of PM4.0 (**d**) and PM10 (**e**) at a concentration of 150 μg/mL.

The previously presented BLM experiments provided new insights into the interactions between pure lipid bilayers and particulate matter. To examine the effects of PM on ion channels, the gramicidin A channels were incorporated into the lipid bilayers. The representative ionic current flow through lipid bilayers before and after the incorporation of gramicidin A channels are presented in Figure 2b. Changes in ionic currents after the addition of PM were analyzed via the calculation of ionic current area, as described previously (see Figure 2c and Section 2). Figure 5a presents the current recordings of gA channels alone (control) and in the presence of PM samples. The current–voltage relationship clearly indicates the inhibition of the gA channel by 150 μg/mL PM4.0 (Figure 5b) and PM10 (Figure 5c) with a more potent PM10 inhibitory effect. But, the analysis of the reversal potential, which was calculated based on the curve intersection with the voltage axis, indicated no significant changes. Additionally, the current amplitude presented in Figure 5d,e made it possible to calculate the conductance. It turned out that PM4 and PM10 presence did not affect the gA channel conductance at a significant level compared to the control. In Table 1, the conductance and reversal potential of the gramicidin A channel in the control condition and in the presence of 150 μg/mL PM4.0 or PM10 have been summarized.

## 4. Discussion

The negative impact of particulate matter (PM) on living organisms has been extensively reported [6,25]. It has already been shown that PM with high contents of heavy metals, such as cadmium and lead, induces large drops in cell viability, increases DNA damage, and drives redistribution among morphological subtypes [26]. Also, our data strongly indicate that PM4.0 and PM10 at a 50 µg/mL concentration have a negative, time-dependent impact on living cells. The presence of PM in the cell culture medium resulted in an increased number of dead cells and cellular debris (Figure 1).

However, there are still very little data describing the effect of PM on ion transport and the bioelectrical properties of biological membranes. Therefore, the aim of our studies was to investigate the changes in the bioelectrical properties (such as electric capacitance and ionic currents) of biological membranes caused by the presence of PM.

One of the best methods for the evaluation of membrane quality changes and their bioelectric properties is the black lipid membrane technique. In recent decades, this well-established method has been extensively used to study a plethora of antibacterial copolymers, ionophoric agents and ion channels, including those found in intracellular membranes [16,27,28]. To our knowledge, a study on the interactions between PM and biological membranes has not been reported yet, leaving some open questions:

(1) Does PM permeabilize biological membranes?
(2) How does the presence of PM affect the electrical capacity of biological membranes?
(3) Does PM regulate the flow of ions through ion channels?

It is known that a sudden increase in membrane permeability usually leads to cell death [29]. Our data from ion-current flow recordings show that the presence of PM4.0 and PM10 at various concentrations does not lead to the permeabilization of lipid membranes. Moreover, the presence of PM4.0 and PM10 reduced the basal ionic currents flowing through the lipid bilayers when negative potentials were applied (Figure 3), with a higher current reduction observed for PM10. On the contrary, when positive potentials were applied, a strong and statistically significant increase in ionic currents passing through the lipid bilayers was observed only in the presence of PM4.0. One of the possible explanations of this phenomenon may be the interaction between PM and phospholipids building up the lipid bilayer. These interactions may affect the physico-chemical properties of the membranes such as fluidity and stiffening, leading to changes in biophysical properties.

Biological and artificial membranes have a thickness of about 7.5 nm [30]. The incorporation of 4 or 10 µm diameter PM into the membranes should result in a change in the lipid bilayer thickness and in the value of electrical capacitance. Our membrane capacitance measurements have shown no statistically significant changes between pure and PM-treated lipid bilayers. However, the tendency of capacity decrease caused by the presence of PM was observed (Figure 4). These observations may suggest that particulates do not incorporate into the membranes as this would result in a change in the bilayer thickness leading to electric capacitance change. It is therefore possible that the PM either passes through the membranes or deposits on their surface (as depicted on Figure 1).

To investigate the effect of PM on ion channels, the well-established, ion-channel-forming peptide gramicidin A (gA) was used [31]. Our results clearly show that both 150 µg/mL PM4.0 and PM10 inhibit the gA ionic currents; however, the reason for this phenomenon remains unclear. With some degree of certainty, we can rule out the effect on channel gating. Our results show that the conductance and reversal potential of gA channels were not affected by the PM. It was recently reported that urban particulate matter does not lead to dysfunction of the CFTR channel [3]. On the other hand, there are also reports showing the activatory effect of PM on ion channels [32]. Our results also show that the presence of PM does not affect the amplitude of single gA channel opening. However, the measured currents were smaller. This could be explained by the impairment in the ion transportation mechanism of the active channel. Another explanation for these observations is the possible effect of the PM charge on gA channel conductance. The particulate matter net charge was found to be electronegative [33]. The deposition of charged molecules on the lipid bilayers might affect the channel conductance, as described previously [33]. Results similar to ours were obtained by Gurnev and Bezrukov who showed that gA channels become rectifying in the presence of trivalent cations, which was explained by the asymmetrical membrane charging [34].

The other explanation assumes an indirect interaction (macromolecule–lipid–protein) between PM and gA proteins [35]. Therefore, it is possible that by an interaction with lipids, PM indirectly affects the gA channels. Since the functional gA channels are formed by the transbilayer dimerization of two non-conducting subunits [18], lower ionic currents through gA channels observed in the presence of PM might result from a lower amount of functional channels formed. The interaction of PM with membranes may also change the membrane stiffness and, in turn, affect the frequency of channel openings. It is noteworthy to mention that PM has pro-oxidative properties and can lead to lipid peroxidation. The products of lipid peroxidation, in turn, have the ability to interact with proteins affecting their structure and activity [36].

## 5. Conclusions

The presence of PM did not lead to lipid membrane permeabilization and had no significant effect on membrane capacitance. These results, together with the observations of basal ionic current reduction at negative potentials and increase at positive ones, suggest that PM most likely deposits at the bilayer surface. Taking the above-mentioned information together, our results show that PM does not significantly alter the basic membrane physiology. Interestingly, we also show that the presence of PM inhibits the gA ionic currents, however with no effect on channel conductance and reversal potential. These observations suggest that PM prevents the formation of functional ion channels formed by gA.

The obtained results, as well as a few publications, suggest that particulate matter may affect proteins that are often important components of cell membranes. This indicates the need for further work on the impact of urban dust on proteins, e.g., cell membrane channels or those present in intracellular organelles.

## Figures and Tables

**Table 1:** Biophysical properties of the gramicidin A channel in the control condition and in the presence of 150 μg/mL PM4.0 or PM10.

Condition	Conductance (pS) ^1^	Reversal Potential (mV) ^2^
Control	31.7 ± 0.5	26.1 ± 0.7
PM4.0	29.1 ± 0.2	23.9 ± 0.9
PM10	30.1 ± 0.6	24.5 ± 0.8

^1,2^ Changes in conductance and reversal potential (Nernst potential) were not statistically significant.

## Data Availability

Not applicable.
